# Molecular Aspects of Hematological Malignancies and Benign Hematological Disorders

**DOI:** 10.3390/ijms24129816

**Published:** 2023-06-06

**Authors:** Amelia Maria Găman

**Affiliations:** 1 Department of Pathophysiology, University of Medicine and Pharmacy of Craiova, 200349 Craiova, Romania; gamanamelia@yahoo.com; 2 Clinic of Hematology, Filantropia City Hospital, 200143 Craiova, Romania

Hematology represents a dynamic specialty in clinical medicine that requires solid knowledge of normal and pathological hematopoiesis, cytomorphology, pathology, immunology, genetics and molecular biology. Advanced laboratory techniques have allowed for an accurate diagnosis, prognosis and monitoring of both hematological malignancies and benign hemopathies, as well as for the application of targeted therapies that have revolutionized the management and therapeutic approach of patients diagnosed with blood disorders. The practice of clinical hematology would not be possible without the laboratory data provided by experts in the fields of cytomorphology, flow-cytometry, pathology and immunohistochemistry, cytogenetics, molecular biology, etc. In addition, the interplay between the clinical and laboratory assessment of blood disorders has further been refined by the introduction of omics technology. Today, the employment of next-generation sequencing [1], digital PCR [2], liquid biopsy [3] and even CRISPR [4] are slowly paving the future of new strategies in the molecular investigation of benign and malignant hematological disorders. Thereby, a dedicated Special Issue to tackle recent advances in the study of molecular aspects of hemopathies was warranted. 

The present Special Issue of the journal was focused on the molecular mechanisms that drive benign and malignant hematological disorders, apparently with a particular emphasis on aggressive lymphomas. In total, seven papers were accepted for publication, of which four were original research studies and the remaining three were narrative reviews. The aforementioned manuscripts have already attracted the attention of the international scientific community, having been cited as of today 13 times. Herein, we provide an overview of the articles accepted for publication in this Special Issue of International Journal of Molecular Sciences.

Burkitt lymphoma, an aggressive form of B-cell non-Hodgkin’s lymphoma, is linked with a specific genetic alteration, i.e., t (8;14), a reciprocal translocation that involves the *c-MYC* oncogene on chromosome 8 and the *IGH* locus on chromosome 14, respectively. In a vast majority of cases, patients also test positive for infection with a gammaherpesvirus, namely the Epstein-Barr viral agent. In their in vitro assessment, Benedetti et al., examined the impact of simultaneous inhibition of the unfolded protein response IRE1α/XBP1 axis and of poly (ADP-ribose) polymerase on cellular models of Burkitt and primary effusion lymphoma. The researchers demonstrated that the co-inhibition of the aforementioned targets might emerge as a promising therapeutic approach in the management of c-MYC- and EBV-associated lymphomas [5]. 

Acute myeloid leukemia remains one of the most aggressive blood cancers and is linked with a dismal prognosis, despite significant discoveries in the biology of this disease, of target therapies and breakthroughs in bone marrow transplantation and cancer immunotherapy [6,7]. Thereby, the identification of novel pathogenetic mechanisms involved in its development and evolution are of paramount importance in the active search for curative approaches. The assessment conducted by Cela et al., employed proteomics techniques to investigate the role of nucleostemin in a cellular model of NMP1-mutated acute myeloid leukemia. Their findings suggest that nucleostemin is involved in the process of DNA repair in leukemic cells [8].

Primary mediastinal B-cell lymphoma is yet another challenging entity in the field of aggressive non-Hodgkin’s lymphomas associated with various genetic alterations. Many of its molecular drivers, however, remain evasive despite significant advances that have been carried out in the management of blood cancers. An in vitro investigation carried out by Liu et al., was able to generate a complex characterization of the V184D, R221G, and G259V human protein tyrosine phosphatase 1B mutants, revealing a potential role of this enzyme in lymphomagenesis [9].

Primary effusion lymphoma pertains to the group of aggressive B-cell non-Hodgkin’s lymphomas and has been associated with the infection with the Human Herpesvirus 8 (HHV-8), the viral agent involved in the development of Kaposi’s sarcoma. Nevertheless, treatment strategies directed against this challenging malignancy remain unsatisfactory and require further investigation. Consequently, Gonnella et al., evaluated the potential benefits of dimethyl fumarate administration in a cellular line of primary effusion lymphoma, as well as its impact on fresh lymphoma cells collected from murine models. Their results point out that dimethyl fumarate exerts anti-inflammatory and antioxidant properties and inhibits key molecules involved in the pathobiology of this hematologic malignancy [10].

Our current understanding of the complex architecture of the hematopoietic stem cell niche and the molecular events that take place in this complicated *milieu* has been enabled by research conducted on zebrafish models. Faisal et al., provide a comprehensive overview of the interactions between hematopoietic stem cells, hematopoietic progenitor cells and the bone marrow microenvironment in zebrafish, with a peculiar focus on epigenetic factors, cell signaling, the role of RNA-binding proteins, as well as novel technologies employed for the characterization of the hematopoietic stem cell niche, including CRISPR/Cas9-based bioinformatics approaches [11].

In their manuscript, Barreno-Rocha et al., summarize the available literature on the crosstalk between lipids, antiphospholipid antibodies and other autoantibodies and hematological malignancies. Their comprehensive immunology analysis points out that autoantibodies can be detected in a wide range of blood cancers, including Hodgkin’s lymphoma, non-Hodgkin’s lymphoma, multiple myeloma, as well as acute and chronic leukemias [12].

Last but not least, this Special Issue touches upon the intricate field of myelodysplastic syndromes. In their top-notch literature analysis, Awada et al., discuss the available prognostic indices employed in the characterization of myelodysplastic syndromes and highlight the importance of personalized risk schemes and the applicability of artificial intelligence in the development of prognostication models based on data derived from genomic studies [13].

To sum it up, we believe that the publication of this Special Issue has shed light on the intricate field of molecular hematology.
